# Long COVID demographic and secondary care referral characteristics in primary care: analysis of anonymised primary care data from a multiethnic, deprived urban area in the UK

**DOI:** 10.1038/s41533-024-00385-8

**Published:** 2024-09-30

**Authors:** Martin Chapman, Stevo Durbaba, Florence Tydeman, Matt Friend, Laura Duly, Julie Moore, Vasa Curcin, Yanzhong Wang, Caroline J. Jolley, Georgios Kaltsakas, Trudie Chalder, Nicholas Hart, Mark Ashworth

**Affiliations:** 1 Department of Population Health Sciences, King’s College London, London, UK; 2 South East London Long COVID Programme, Guy’s & St Thomas’ NHS Foundation Trust, London, UK; 3 Centre for Human and Applied Physiological Sciences, King’s College London, London, UK; 4 Department of Respiratory Medicine, King’s College Hospital NHS Foundation Trust, London, UK; 5 Lane Fox Clinical Respiratory Physiology Research Centre, Guy’s & St Thomas’ NHS Foundation Trust, London, UK; 6 Department of Psychological Medicine, Institute of Psychiatry, Psychology & Neuroscience, King’s College London, London, UK

**Keywords:** Epidemiology, Health services, Respiratory signs and symptoms

## Abstract

Once the nature and number of patients with Long COVID was more fully understood, UK secondary care developed services to investigate, treat and support these patients. We aimed to identify evidence for demographic health inequalities based on general practitioner (GP) Long COVID referrals to available secondary care services. Despite Long COVID demographics broadly reflecting the multiethnic and socially disadvantaged profile of the study population, we found that secondary care referral was mainly focussed on older age patients and those born in the UK with co-morbid anxiety; although co-morbid diabetes was associated with reduced referrals.

## Introduction

Healthcare services worldwide experienced overwhelming demand for the acute care of patients with COVID-19, particularly in the early stages of the pandemic. Soon after clinicians became more familiar with the often intensive healthcare demands for acute management, evidence slowly accumulated of characteristic long-term sequelae^1^. The term ‘Long COVID’ (often now termed ‘Post Covid Condition’ or PCC) was applied to this constellation of symptoms as early as Spring 2020, with the term coined by people experiencing these symptoms^2^. Early attempts were made to create a definition of Long COVID in patients who had ‘survived but not recovered’ based on specified criteria but duration was not included^2^. In December 2022, the WHO published a Factsheet based on global consensus defining Long COVID as the persistence of symptoms for at least 3 months^3^. This report highlighted high prevalence, with studies showing 10-20% of people with acute COVID-19 (even those mildly infected) would go on to develop Long COVID.

Few studies have reported on the characteristics of Long COVID patients referred by their general practitioners (GPs) to available Long COVID secondary care services^4^. Our study aim was firstly to characterise the demographic features of Long Covid in a primary care population and secondly, to determine the features of Long Covid patients referred to secondary care services.

## Methods

### Data source

Data was extracted from an anonymised primary care database, Lambeth DataNet, containing SNOMED-CT coded data^5^ derived from the electronic health records of patients registered at general practices in one inner-London borough (Lambeth) between 01/04/2019 and 30/06/2023, *n* = 581811^6^. Data extraction was conducted on 6th July 2023. Lambeth is an inner city, multiethnic borough with high levels of social deprivation. All patients are included within the database with the exception of those with ‘informed dissent’ codes in their notes (3.6% of the currently registered population). Only coded data is captured, not narrative text data recorded by the GP. As a result, prevalence estimates are likely to be an underestimate.

### Study population

The primary care records of 1188 patients with SNOMED-CT codes for Long COVID were identified on the basis of one or more relevant SNOMED-CT codes in their records. This represents 0.36% of the adult population.

### Statistical analysis

We performed descriptive, univariable and multivariable analyses on our study population. For our analysis, we used demographic variables age, sex, ethnicity, country of origin, deprivation (IMD 2019 score), English language preference and locality; and clinical variables BMI, COVID (suspected/confirmed) and the presence of six long-term conditions (LTCs): anxiety, depression, chronic pain, diabetes, obesity and morbid obesity^7^. These six LTCs were selected by two of the clinical co-authors as the LTCs most likely to be stronger determinants of both Long Covid diagnosis and referral in primary care (M.A.: primary care clinician; N.H.: secondary care clinician). Independent variables were added to a binary logistic regression model to determine the association with secondary care referral.

### Ethical approval

This study was exempt from ethical committee approval and the requirement to obtain individual patient consent. Exemptions for UK research studies using anonymised primary care data apply to studies fulfilling requirements specified by the national body, the NHS Health Research Authority (HRA).

## Results

### Univariable analysis

The mean age of the Long Covid sample was 45.6 years (standard deviation: 14.6; 5th and 95th centiles: 25.0, 75.0). The mean Long COVID referral rate was 42%. Higher proportions were found in patients who were female, of White ethnicity and born in the UK (Table 1).

**Table 1:** Demographic variables: proportions of sample patients and patients referred to secondary care.

Variable	Sample *n* = 1188 (%)	Referred (%)
Male gender	38%	39%
Female gender	62%	44%
White ethnicity	60%	47%
Black ethnicity	18%	37%
Mixed ethnicity	6%	31%
Asian ethnicity	10%	30%
Born in UK	61%	50%
Not Born in UK	39%	33%
Most deprived quintile	16%	39%
Least deprived quintile	19%	43%
Anxiety	47%	47%
Chronic Pain	41%	46%
Depression	37%	48%
Diabetes	9.2%	33%
Obesity	32%	44%
Morbid obesity	9.2%	41%

### Multivariable analysis

The results of our multivariable analysis are shown in Table 2. Here, when accounting for the impact of multiple variables on referral rates, we observe similar associations to our univariate analysis but with only some variables emerging as significantly associated with secondary care referral following multivariable adjustment.

**Table 2:** Associations between secondary care referral and demographic and clinical predictor variables.

Variable	Adjusted odds ratio (AOR)	95% Confidence interval, AOR	Significance, *P*
Age (years)	1.01	1.00, 1.03	0.022*
Gender (female)	1.36	0.99, 1.88	0.062
White ethnicity	REFERENCE GROUP		
Black ethnicity	0.59	0.33, 1.06	0.078
Asian ethnicity	0.61	0.30, 1.24	0.171
Mixed ethnicity	0.40	0.17, 0.92	0.030*
Other ethnicity	0.67	0.28, 1.61	0.368
Unknown ethnicity	0.89	0.51, 1.54	0.669
English Language preference	0.84	0.50, 1.39	0.493
UK Country of origin	2.18	1.33, 3.57	0.001***
Locality: north	REFERENCE GROUP		
Locality: south east	0.78	0.52, 1.17	0.231
Locality: south west	1.00	0.69, 1.46	0.998
Least deprived quintile	REFERENCE GROUP		
2nd least deprived quintile	0.62	0.11, 3.49	0.588
3rd least deprived quintile	0.60	0.11, 3.30	0.554
4th most deprived quintile	0.69	0.13, 3.81	0.670
Most deprived quintile	0.71	0.13, 3.94	0.695
Acute Covid: suspected/confirmed	0.93	0.66, 1.31	0.670
Anxiety	1.47	1.04, 2.08	0.028*
Chronic Pain	1.05	0.72, 1.52	0.808
Depression	0.97	0.67, 1.42	0.880
Diabetes	0.49	0.26, 0.94	0.032*
Obesity	1.02	0.69, 1.50	0.928
Morbid Obesity	1.18	0.62, 2.26	0.614

^*^P < 0.05; **P < 0.01; ***P < 0.001.

## Discussion

### Main findings

The key drivers to secondary care referral in our study were: older age (increased referrals); mixed ethnicity (reduced referrals); born in the UK (increased referrals); anxiety (increased referrals); diabetes (reduced referrals). Although Black ethnicity (reduced referrals) and female gender (increased referrals) had large AORs, these were uncertain given our sample size (not statistically significant).

### Comparison with literature

In March 2023, the Office for National Statistics estimated that 1.9 million people in the UK (2.9% of the population) were experiencing self-reported Long COVID symptoms^8^. In terms of symptoms, fatigue was the most commonly reported symptom (72%) followed by difficulty concentrating (51%), muscle ache (49%) and shortness of breath (48%). These estimates were based on a household survey sent to almost 270,000 individuals and face-to-face interviews of selected samples. In a population study from Scotland, Long Covid prevalence, after adjustment for confounding, was 6.6% at 6 months in those with a history of acute infection^9^.

Studies of community prevalence are always likely to reveal higher prevalence estimates than studies conducted in primary care as a proportion of patients seek alternatives to primary care such as self-management approaches^10^. However, doubts have been raised about the clinical coding of Long COVID in UK primary care, suggesting substantial under-coding^11^. Based on a study of OpenSAFELY primary care data, the recorded prevalence of Long COVID in the London region was 55.6 per 100,000 (0.06%), much lower than in community estimates with just over a quarter of all practices reporting no diagnostic codes for Long COVID codes implying lack of coding rather than zero prevalence. In our study of an inner London, deprived and multiethnic borough, the prevalence was 0.36% and all practices had Long COVID codings. This higher rate than the London average probably represents higher acute COVID infection rates associated with the known acute Covid risk factors of social deprivation and multi-ethnicity^12^.

### Strengths and weaknesses

Our findings relate to the secondary care referral characteristics of patients with a primary care diagnosis of Long COVID. The demographic characteristics of those patients referred with Long COVID may suggest health inequalities based on unequal referral thresholds for different sectors of the population. Alternatively, they may represent gradations of Long COVID severity or variable symptom patterns with some symptoms more likely than others to trigger referral^13^. This study based on Long COVID and co-morbidity diagnostic data was unable to determine disease severity and it is possible that, for example, Long COVID severity was greater in older patients with anxiety.

In our analysis, older age was a predictor of referral. However, age was skewed with small numbers of much older age Long Covid patients which may have distorted the regression model, resulting in an overestimate of the effect of age. Further research is needed on the differential effect of age categories, although our sample size in older patients was not sufficient for such analysis.

We included a variable in the regression analysis, ‘acute Covid: suspected/confirmed’. This variable was intended as a proxy indicator to capture those patients more engaged with primary care, since confirmation of an acute Covid diagnosis often relied on patients notifying their GP of the results of self-testing at home. However, this variable was not a significant predictor of Long Covid referral implying that help-seeking for acute Covid was not associated with secondary care help-seeking.

There may also have been differences in ‘coding threshold’ whereby GPs varied in their readiness to attach a Long COVID diagnostic label to a patient’s symptoms. However, our data shows no significant differences in referral thresholds between the three localities included in our study, implying few geographical differences between practices.

The overall prevalence estimate for Long COVID depended on identifying relevant SNOMED-CT codes, ensuring that no commonly used codes were omitted. Patients were only included if they had a formal diagnostic coding, and symptom coding based on the most commonly recorded Long COVID symptoms^14^ was not included. This could have substantially increased the estimated prevalence although records of symptom duration are less reliably obtained from electronic health records and therefore this was not included in our study.

### Implications

Long COVID patients seen in secondary care services may be a selected population unrepresentative of the demographic characteristics of Long COVID in primary care. Epidemiological Long COVID studies need to consider a broader remit than secondary care populations. Further study is needed on the epidemiological characteristics of Long Covid in primary care, particularly in areas where our study may not translate so effectively, such as suburban or rural communities.

## Data Availability

The datasets analysed during the current study are not publicly available due to the terms of primary care data access. Long Term Condition SNOMED-CT codes are available on GitHub.
